# Advances in ovarian follicle culture systems: exploring the interplay between cells, matrix, and ovarian architecture

**DOI:** 10.1590/1984-3143-AR2025-0066

**Published:** 2025-08-14

**Authors:** Alberto Maria Luciano, Noemi Monferini, Ludovica Donadini, Pritha Dey, Fernanda Fagali Franchi, Valentina Lodde, Federica Franciosi

**Affiliations:** 1 Reproductive and Developmental Biology Laboratory – ReDBioLab, Department of Veterinary Medicine and Animal Sciences, University of Milan, Milan, Italy; 2 Center for Reproductive Biotechnology and Cryobanking, University of Milan, Milan, Italy

**Keywords:** preantral follicles, culture system, 3D, ovary, scaffold

## Abstract

The ability to develop oocytes from the earliest follicular stages through maturation and fertilization in vitro would revolutionize fertility preservation in human medicine and animal breeding. Instead, current assisted reproductive technologies rely only on a limited portion of the female gamete reserve, corresponding to the antral population, while the preantral follicle reserve remains unexploited, mainly due to a lack of knowledge regarding the mechanisms that guide preantral follicle differentiation and folliculogenesis in vitro. This review highlights the efforts made thus far and suggests an approach to studying the mechanisms and ovarian environment to enhance preantral follicle culture systems.

## Introduction

Assisted reproductive technologies (ARTs) are fundamental for fertility preservation. However, current ART procedures rely only on a limited portion of the female gamete reserve, specifically the antral follicles. In contrast, the preantral follicle population, which represents 99% of the reserve and comprises primordial (PMF), primary (PF), and secondary (SF) follicles, remains unexploited (Telfer et al., 2023). To date, cryopreservation of ovarian cortical tissue containing preantral follicles has emerged as a critical, albeit temporary, solution for preserving this valuable biological material (Bolton et al., 2022). Practical applications that benefit from the potential of ovarian cortex fragments in cryobanks remain limited (Bolton et al., 2022; Hussein et al., 2020; Tao and Del Valle, 2008). This limitation arises from the absence of an efficient and reliable in vitro culture system capable of developing the enclosed preantral follicles (Dey et al., 2024a). The lack of a culture system results from limited knowledge of the physiological conditions that must be recapitulated in vitro to develop oocytes enclosed in early-stage follicles into mature, fertilizable gametes (Dey et al., 2024a; Frost and Gilchrist, 2024). Implementing a suitable animal model is essential to uncover the mechanisms driving folliculogenesis and to design an efficient culture system that fills this gap (Telfer et al., 2023).

**Figure 1:**
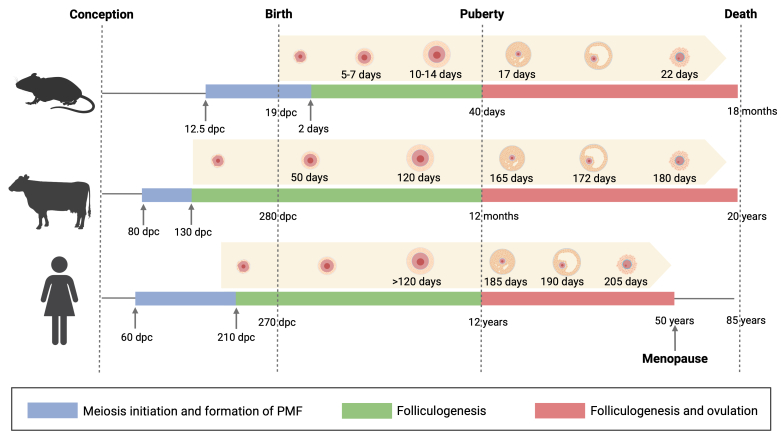
Comparison of the oogenesis and folliculogenesis timeline in mouse, bovine, and humans from conception to death. Ages are indicated under the bars. Before birth, ages are indicated as days post conception (dpc). The developmental phases are described in the figure legend. The boundary between the blue and green bars corresponds to the stage at which most PMF have been formed. The boundary between the green and red bars corresponds to the first occurrence of complete follicular development, culminating in ovulation. The complete exhaustion of the reserve occurs only in humans and certain primates, likely associated with their long lifespan. The upper orange arrows show the developmental timing required from each of the three species for a PMF to differentiate until ovulation (Fair and Lonergan, 2023; Ge et al., 2019; Gougeon, 1996; Hoage and Cameron, 1976; McGee and Hsueh, 2000; Monniaux et al., 2014; Richard et al., 2023). Created in BioRender by Monferini (2025).

Research models provide a conceptual framework upon which specific hypotheses can be built and tested, allowing for the extension of concepts across different species. As the most commonly used animal model in preantral follicle culture, mice are prevalent in studying physiology and biological processes because they are easy to manipulate and allow for the creation of genetically modified individuals (Ménézo and Hérubel, 2002). However, due to intrinsic biological differences in PMF formation, the timing and the mechanisms of folliculogenesis, mice cannot recapitulate folliculogenesis in higher-order mammals (Frost and Gilchrist, 2024). PMFs in mice develop postnatally and are activated in two distinct waves. The first wave synchronously activates PMFs in the medullary region that do not progress to the ovulatory stage. The second wave gradually develops PMFs in the cortical region, which then contribute to the formation of mature oocytes capable of undergoing fertilization (Zheng et al., 2014). These mechanisms differ in monotocous mammals, where PMFs form during fetal life (Figure 1) (Denicol, 2024; Ge et al., 2019; Monniaux et al., 2019; Santos et al., 2013).

After their formation, PMFs undergo a specific period of development. In the murine model, folliculogenesis is estimated to last approximately 30 days from the PMFs to the early antral follicle (EAF) stage. In higher-order mammals, this process is significantly slower (McGee and Hsueh, 2000). In humans and cattle, it takes over 120-180 days for an activated PMF to progress to the EAF stage, making the bovine a more comparable model to humans (Campbell et al., 2003; Fair and Lonergan, 2023; Gougeon, 1996; McGee and Hsueh, 2000; Sirard, 2017). Moreover, it is essential to consider additional factors that influence ovarian dynamics beyond PMF development and can significantly impact overall follicle growth (Zhang et al., 2024). Regarding the surrounding environment, human and bovine species share similar tissue and follicle organization within the ovary (Kagawa et al., 2009), and both monotocous species, compared to mice, have similar hormonal regulation and follicular physiology (Adams and Pierson, 1995; Baerwald et al., 2003; Baerwald et al., 2012; Sirard, 2017). These findings further emphasize the suitability of the bovine model for studying fertility preservation, underscoring its potential as a model for aging research (Malhi et al., 2005) and for developing applications in human reproductive medicine (Sirard, 2017).

## The ovarian reserve

**Figure 2:**
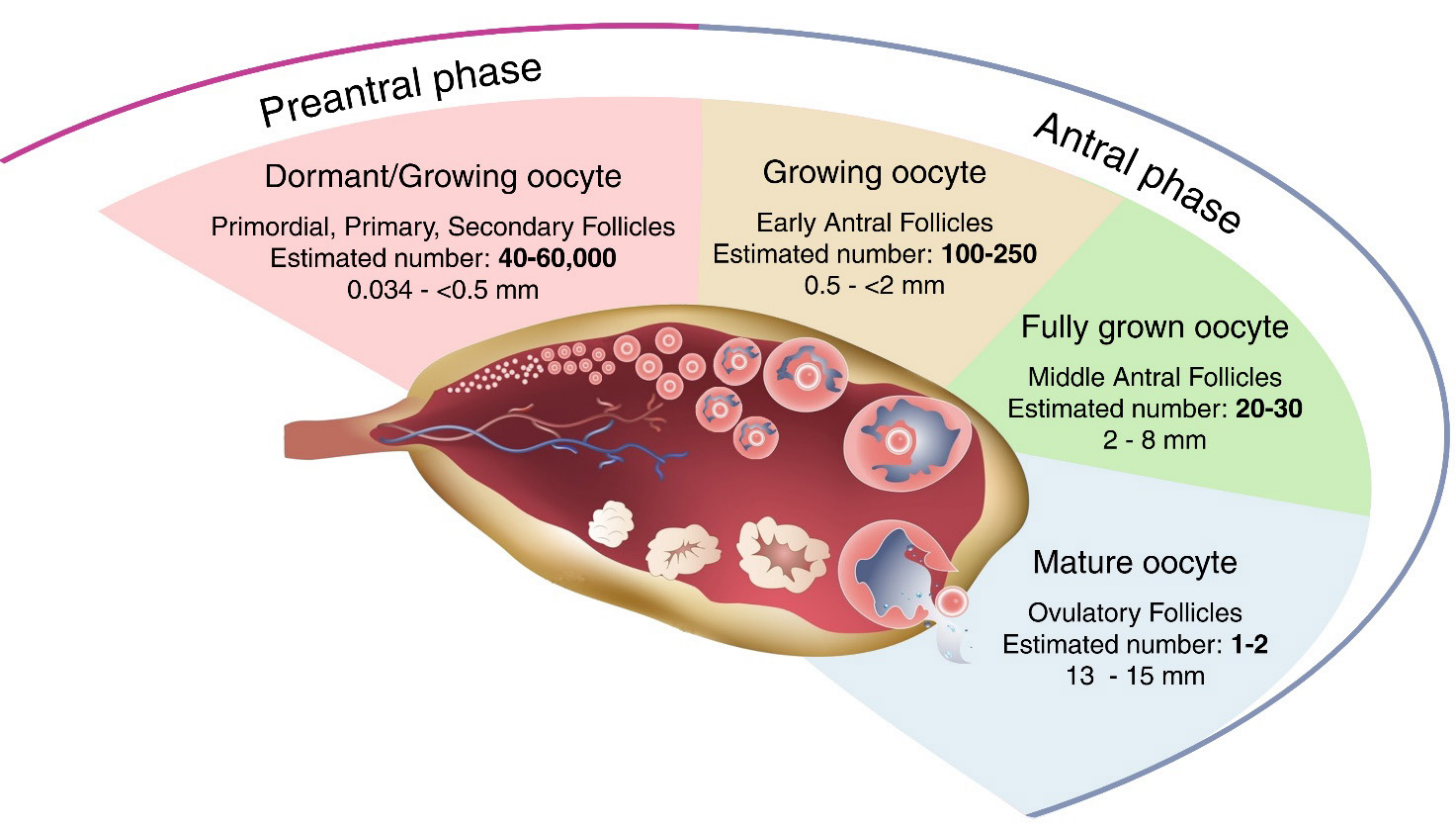
Extrapolation of the bovine follicle reserve at a given time in a cycling ovary. Based on Erickson (1966), Lussier et al. (1987), Modina et al. (2014) and Silva-Santos et al. (2011). Created in BioRender by Monferini (2025).

The ovarian reserve resides within the ovarian cortex and is primarily comprised of PMF, the earliest stage of the preantral follicles (Figure 2) (Erickson, 1966; Modina et al., 2014; Telfer et al., 2023).

The number of follicles in the ovaries of mammals varies remarkably between species and even among individuals of the same species (Lambalk et al., 2004). Due to these species-specific differences, this section compares the ovarian reserve sizes of two monotocous species (humans and bovine) while excluding polytocous species, such as the mouse, which may display a different ovarian reserve pattern (Findlay et al., 2015). Based on the limited available histological analyses of whole ovary counts at birth, the ovarian reserve in humans ranges from 350,000 to 1,100,000 (Forabosco and Sforza, 2007; Gougeon et al., 1994), while in cattle, it ranges from approximately 14,000 to 250,000 (Erickson, 1966; Silva-Santos et al., 2011). During puberty, the reserve is approximately 100,000 in women and 84,000 in heifers, respectively, with a significant decline in the fourth year of life for cows (Dey et al., 2024b; Erickson, 1966; Modina et al., 2014; Monferini et al., 2024; Silva-Santos et al., 2011; Telfer et al., 2023; Wallace and Kelsey, 2010), corresponding to the decline observed in women between 31 and 35 years (Faddy et al., 1992; Gougeon, 2004).

The ovarian follicle reserve is non-renewable, as far as is known, and requires regulation throughout the reproductive lifespan of mammals. Unlike other cell types in the body that can regenerate or increase in number through mitotic cycles, the number of oocytes is fixed at birth and gradually declines over a woman’s lifetime. As females age, both the quantity and quality of their oocytes decline (Brieño-Enriquez et al., 2022; Wang et al., 2023). The natural depletion process means that, over time, fewer oocytes remain available for recruitment and selection to mature to the stage where they can be successfully fertilized. This decline in the quantity and quality of oocytes significantly impacts fertility and reproductive health (Telfer et al., 2023).

To ensure its longevity, survival, and quality, the ovarian reserve is governed by complex mechanisms that control PMF quiescence and recruitment (Dri et al., 2021). Throughout their entire reproductive lifespan, PMFs exhibit three distinct fates: (i) they can remain quiescent, surviving for varying lengths of time during reproductive life; (ii) they may be activated and differentiate into the pool of growing follicles, later followed by either atresia or ovulation; (iii) they may undergo cell death mechanisms, directly after the quiescent stage, contributing to female reproductive aging (Hansen et al., 2008; McGee and Hsueh, 2000). Until now, very little is known about the mechanisms involved in these three destinies, with most regulatory insights stemming from gene editing in mice.

## Isolated and in situ follicle culture: limitations in supporting preantral follicle viability

Preantral class or gonadotropin-independent phase comprises three major follicular stages: PMF, PF, and SF (Fair et al., 1997; Monferini et al., 2024; Williams and Erickson, 2000). To support follicle development in vitro, it is essential to understand the specific growth requirements associated with each stage of folliculogenesis. Culturing isolated follicles offers a strategy to minimize the variability between PMF, PF, and SF. Working with a homogeneous population of follicles at the same developmental stage enables more precise control of the culture environment to meet their specific needs. However, the isolation method is critical, as it can strongly influence follicle viability and survival outcomes (Dey et al., 2024a; Telfer et al., 2000; Wandji et al., 1996). The culture of isolated follicles in a 2D system results in low maintenance of follicle viability (Dey et al., 2025; Gutierrez et al., 2000), potentially because cell polarity, cellular morphology, and gene expression may not accurately reflect in vivo conditions (Jensen and Teng, 2020). The unnatural geometry of 2D culture disrupts cell-cell communication, inducing granulosa cells to break through the basement membrane, migrate away from the oocyte, and adhere to the surface of the culture dish (Cortvrindt et al., 1996; Gargus and Woodruff, 2021; Kreeger et al., 2006). The intimate relationship between the oocyte and granulosa cells is fundamental to the development of a high-quality oocyte (Moor et al., 1998).

**Figure 3:**
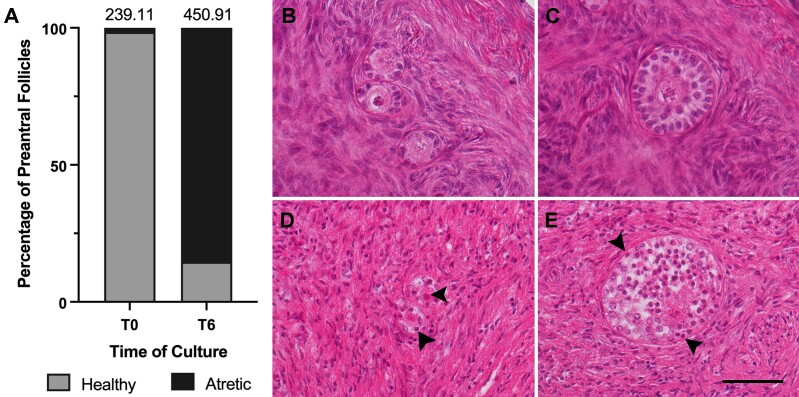
Preantral follicles’ viability before (T0) and after culture (T6). Preantral follicles were cultured through in situ culture of cortical fragments obtained using biopsy punches (KAI Medical, Solingen, Germany) of 2 mm in diameter from ovarian cortex slices 0.5 mm thick. Two fragments for each animal (N=6, age 12-24 months/old) were immediately fixed, while other two fragments were placed in each well of a 24-well cell culture plate in 300 µl of an αMEM-based culture medium, supplemented with 0.1% Bovine Serum Albumin fatty acid free, 1mg/ml r-hInsulin, 0.55 mg/ml hTransferrin, 0.5 µg/ml Sodium Selenite, 10-4 IU/ml r-hFSH, 0.164 mM Penicillin, and 0.048mM Streptomycin (αMEM+). Fragments were cultured for 6 days at 38.5 °C and 5% CO_2_ in air, maximum humidity. Half of the medium was replaced with freshly prepared αMEM+ every second day. To determine follicle viability at T0 and T6, fragments were fixed in Form-Acetic, paraffin-embedded, and stained with Hematoxylin and Eosin. The follicles were counted in serial sections, and the volume of the fragment was obtained using ImageJ. The final number of follicles per mm^3^ was then calculated (as reported in Bjarkadottir et al., 2021; Monferini et al., 2024). The bar graph (A) reports the percentages of healthy and atretic follicles, while at the top of the bar, the total number of follicles/mm^3^ is given; (B) and (C) represent healthy preantral follicles at T0, and (D) and (E) represent atretic preantral follicles at T6. Follicle health was assessed based on the presence or absence of pyknotic granulosa cells or oocyte (black arrowhead), intact basal membrane, oocyte and nucleus, shrinkage of the ooplasm, ooplasm eosinophilia, as described in Monferini et al. (2024). Scale bar = 50 µm.

One apparently effective approach involves culturing follicles within their native cortical environment, also referred to as in situ culture. This method is widely attempted, yet it experiences low efficiency in terms of follicle transition. Moreover, follicular viability is often overlooked in many studies, which consider only the success rate based on healthy follicles, potentially leading to biased or incomplete results. To establish our laboratory benchmarking process, a method by which laboratories compare their performance with similar peer laboratories worldwide, we conducted a comparative study to validate one of the most robust in situ culture systems from the literature (Bjarkadottir et al., 2021). We evaluated the viability of the ovarian reserve and assessed the follicle growth and transition after 6 days of in situ culture (Figure 3). Follicle growth was not observed, instead preantral follicle viability decreased significantly from the non-cultured fragments (T0, 99% healthy follicles) to those cultured for 6 days (T6, >30% healthy follicles), in line with previous in situ culture studies (Bjarkadottir et al., 2021; Walker et al., 2021).

Additional limitations associated with in situ culture include the heterogeneity of the evaluated follicle population and the cortical environment. In particular, accurately assessing changes in follicle density during in situ culture remains challenging due to the unknown initial follicle populations in a given fragment, as the distribution of preantral follicles in the ovary is heterogeneous, leading to a lack of comparability between different biopsies from the same ovary (Poirot et al., 2002; Schmidt et al., 2003). In a debate by Lambalk et al., this issue was raised with a provoking question: *“Is the lack of follicles in a single biopsy, or indeed any number of biopsies, enough to make accurate diagnoses, or are they more misleading than prognosticating?”* (Lambalk et al., 2004). Our experience with morphometric evaluation of the follicle population is consistent with the above statements. What we observed during the counting is that follicle density varied between fragments from the same animal, and follicles also appeared clustered or isolated within the same fragment (data not shown). This limitation can lead to ambiguous interpretations of the results regarding ovarian follicle density. On the other hand, while harnessing the original follicle environment to culture follicles is ideally the most appropriate and preferable way to sustain their growth in vitro, this is not always the case. The tissue loses its properties during culture, and collagen fibers diminish, as well as the number of cells, altering the microenvironment surrounding the follicles (Grosbois et al., 2023). The mechanical compression that the extracellular matrix (ECM) exerts on the PMF to maintain quiescence is lost, leading to activation (Nagamatsu et al., 2019). The spontaneous recruitment of quiescent PMFs into the growing pool during culture may also result from the processing of ovarian cortical tissue prior to culture. Fragmentation disrupts the Hippo signaling pathway, leading to PMF activation through PI3K/Akt upregulation (Masciangelo et al., 2020; Telfer et al., 2023). Additionally, removing a small cortical fragment excludes the population of more advanced-stage follicles responsible for secreting high levels of AMH, a key inhibitor and regulator of follicle growth (Greve et al., 2012; Roness et al., 2013). Spontaneous and massive activation has not been proven to be an effective approach. Dysregulation of homeostasis can harm follicle viability and contribute to “follicle burnout” (Masciangelo et al., 2020; Roness et al., 2013). During in situ culture, atresia affects more than 80% of PMF, likely due to the massive activation and subsequent degeneration of the PMF pool, which damages follicle homeostasis.

## The artificial ovary: a strategy for in vitro follicle culture

The ideal environment for culturing follicles may be in a 3D system that enables the recreation of organ architecture and mechanical maintenance for the follicles, which can be achieved using matrices (reviewed in (Zuccotti et al., 2015)). Matrices are mainly based on alginate, hydrogel, collagen, decellularized matrix, or other gelling agents (Babayev et al., 2022; Belli et al., 2012; Campo et al., 2021; Laronda et al., 2014; McDowell et al., 2024; Pietroforte et al., 2024). The purpose of the matrix is to mimic the structural support provided by the cortical ovarian stroma, exerting mechanical properties that regulate signal transduction during follicle quiescence and recruitment (Fiorentino et al., 2023). Furthermore, these systems, widely used in cancer cell cultures to study mechanisms of tumor progression in vitro, highlight favorable characteristics to maintain proper cell-cell and cell-matrix interactions, preserve morphology, division patterns, cell polarity, nutrient availability, and gene expression similar to in vivo conditions (reviewed in Kapałczyńska et al., 2018).

For preantral follicles, 3D systems are the ideal approach to facilitate the control of culture conditions, potentially creating homogeneous populations of isolated follicles. Their development requires several structural and functional changes, as described in an accompanying paper by our group in this same special issue (Lodde et al., 2025 forthcoming), together with proper gene regulation at the proper follicular stage, establishing a finely orchestrated communication between the germinal (the oocyte) and the somatic (the granulosa cells) compartments (Bonnet et al., 2013; Rodrigues et al., 2021). Oocyte and granulosa cell bi-directional interactions regulate follicle growth in an autocrine and paracrine manner via secreted factors and direct communication, such as through gap junctions (Albertini et al., 2001; Bonnet et al., 2013; Eppig, 2001; Martinez et al., 2023). Paracrine communication within the follicle is regulated by oocyte-secreted factors, such as growth and differentiation factor 9 (GDF9) and bone morphogenic protein 15 (BMP15), and members of the TGFβ superfamily, which positively influence the preantral follicle growth (reviewed in (Knight and Glister, 2006)). Additionally, the most described paracrine communication in preantral follicles, involved in the cooperative interaction of oocytes and granulosa cells, is the KIT ligand, secreted by granulosa cells and playing a role in stimulating PI3K signaling in the oocyte (Li et al., 2020; Yao et al., 2014; Zhang et al., 2014; Zhao et al., 2018). A study in ovine also suggested that secreted factors, such as vascular endothelial growth factor (VEGF), insulin-like growth factor (IGF), and fibroblast growth factor (FGF), may play a potential role in cell-cell communication in preantral follicles during folliculogenesis (Bonnet et al., 2013). Intra-follicle communication also involves physical communication between oocytes and granulosa cells, mediated by gap junctions (Li and Albertini, 2013), which are intercellular channels connecting adjacent membranes that exchange small molecules, such as ions, cyclic nucleosides, metabolites, amino acids, mRNAs, and miRNAs (Anderson and Albertini, 1976; Kidder and Mhawi, 2002; Martinez et al., 2023). Gap junctions, together with adherens junctions, are assembled on prominent microvillous extensions from the surface of granulosa cells that terminate on the oocyte plasma membrane (Li and Albertini, 2013; Motta et al., 1994). This synergy begins at the primordial stage (Kidder and Mhawi, 2002), and the crosstalk must be sustained for a very prolonged time, particularly in the phase of preantral follicle development (Albertini et al., 2001, 2003; Anderson and Albertini, 1976; Carabatsos et al., 1998). In fact, microvillous structures are present already at the primordial and primary follicle stage, where zona pellucida has not been deposited or is fragmented (Fair et al., 1997; Motta et al., 1994). At the secondary follicle stage, the microvillous projection crosses the thin layer of zona pellucida and begins to be referred as transzonal projection (TZP) (Albertini et al., 2001; Motta et al., 1994; Plancha et al., 2005).

The TZPs increase in number and structural complexity as the oocyte grows and are responsible for the direct transport, mediated by proteins, of RNA (Karen Nenonene et al., 2023; Macaulay et al., 2014). Moreover, in more advanced follicle stages, TZPs enhance oocyte developmental potential by modulating the synthesis/stability of specific oocyte transcripts (Crozet et al., 2023), and facilitate the transport of essential metabolites required for the growth of quality oocytes (Robert, 2021), such as cyclic nucleotides, into the oocyte to maintain meiotic arrest until the fully competent stage (Franciosi et al., 2014; Gilchrist et al., 2016; Luciano et al., 2011). However, many other molecules transported through TZP structures remain unknown. Investigating the interactions and regulatory molecules involved in oocyte-granulosa cell connection from the earliest stages of folliculogenesis holds the potential to significantly enhance strategies for the in vitro growth of preantral follicles, thereby supporting oocyte quality.

In concert with the ovarian parenchyma, specifically the ovarian follicles, the stroma also plays an essential role in ovarian function. More recently, the ovarian stroma has emerged as an exciting new frontier for understanding complex ovarian dynamics and its contribution to folliculogenesis (Gargus and Woodruff, 2021; Kinnear et al., 2020). A growing body of interest has been directed toward ECM and stromal cell signaling (Kinnear et al., 2020; Rodgers et al., 2003). The ECM, also defined as “matrisome” following Naba et al.’s classification (Naba et al., 2016), is composed of 49% collagens, 15% glycoproteins, and 7% proteoglycans, forming the protein core matrisome, along with 18% ECM-affiliated proteins, 10% ECM regulators, and 1% secreted factors (Ouni et al., 2019). The ovarian cortex, where PMFs reside in decellularized human and bovine tissue, appears stiff and rich in collagen fibers compared to the less dense medulla region (Chiti et al., 2018; Laronda et al., 2015). The mechanical properties of the ovarian cortex support PMF maintenance through Hippo pathway activity (Kawamura et al., 2013). Moreover, the ECM initiates a signaling cascade for proliferation and differentiation as follicles develop, and acts as a reservoir for growth factors and cytokines (Kinnear et al., 2020). These processes must be finely regulated because the ECM is continuously modified, and each ovulatory event can degrade and remodel the matrix, influencing follicle activation (Zhang et al., 2024). In vitro, it was possible to replicate the mechanical properties of the ECM by culturing preantral follicles in 0.5% or 2% alginate (Hornick et al., 2012; Pietroforte et al., 2024). In particular, isolated PMFs require a higher alginate concentration (2%) to survive and grow in culture (Hornick et al., 2012). SF show better growth in a less dense environment (0.5%), maintaining cell-cell connections (Pietroforte et al., 2024), as follicles grow larger, they physiologically move to the sparser medullary region (Hsueh et al., 2015). Furthermore, with age, the stiffness of the tissue and the molecular composition of the ECM are altered, impairing follicle health (Amargant et al., 2020; Ouni et al., 2020, 2022).

Besides ECM, the ovarian stroma consists of ovary-specific cell types that interact with the follicles (Kinnear et al., 2020). In creating an “artificial ovary”, the stroma-like cells’ contribution must be considered as a source of soluble signals and mechanical cues that, together with ECM, recreate a complex symphony (Kinnear et al., 2020). Co-culture of alginate-encapsulated murine SF (alginate concentration 0.5%) with theca cells and macrophages promotes follicle survival and development due to factors secreted from stromal cells, including several cytokines and growth factors (Tingen et al., 2011). Other reports in humans confirm that supplementing ovarian stroma cells in culture enhances the transition of alginate-encapsulated follicles, enriching the culture medium with specific growth factors and cytokines (Grubliauskaitė et al., 2024). However, in bovine studies, a culture system for preantral follicles, based on poly(ethylene glycol) (PEG) hydrogels with co-encapsulated ovarian stromal cell, follicles did not maintain viability, probably due to the absence of basement membrane binding proteins in the PEG composition, which rendered the cells unable to adhere to the matrix and resulted in self-aggregation (Candelaria et al., 2025). These findings provide novel insights into the 3D culture of preantral follicles; however, inconsistencies suggest that further refinements are necessary. An in-depth investigation of the type and function of the stromal cells is needed to identify their role in follicle development.

## Future perspectives and conclusions

The exploitation of preantral follicles in vitro remains grounded in the mouse paradigm (Eppig and O’Brien, 1996; O’Brien et al., 2003). In humans, using a multi-step culture system that starts from preantral follicles, a few MII oocytes have been achieved (McLaughlin et al., 2018) several years later. Recently, a blastocyst was obtained through the in vitro growth of SF (Guo et al., 2024). Over the years, improvements have been made to enhance culture systems. Nonetheless, we remain far from establishing a system that can effectively grow preantral follicles in vitro (Dey et al., 2024a; Frost and Gilchrist, 2024).

**Figure 4:**
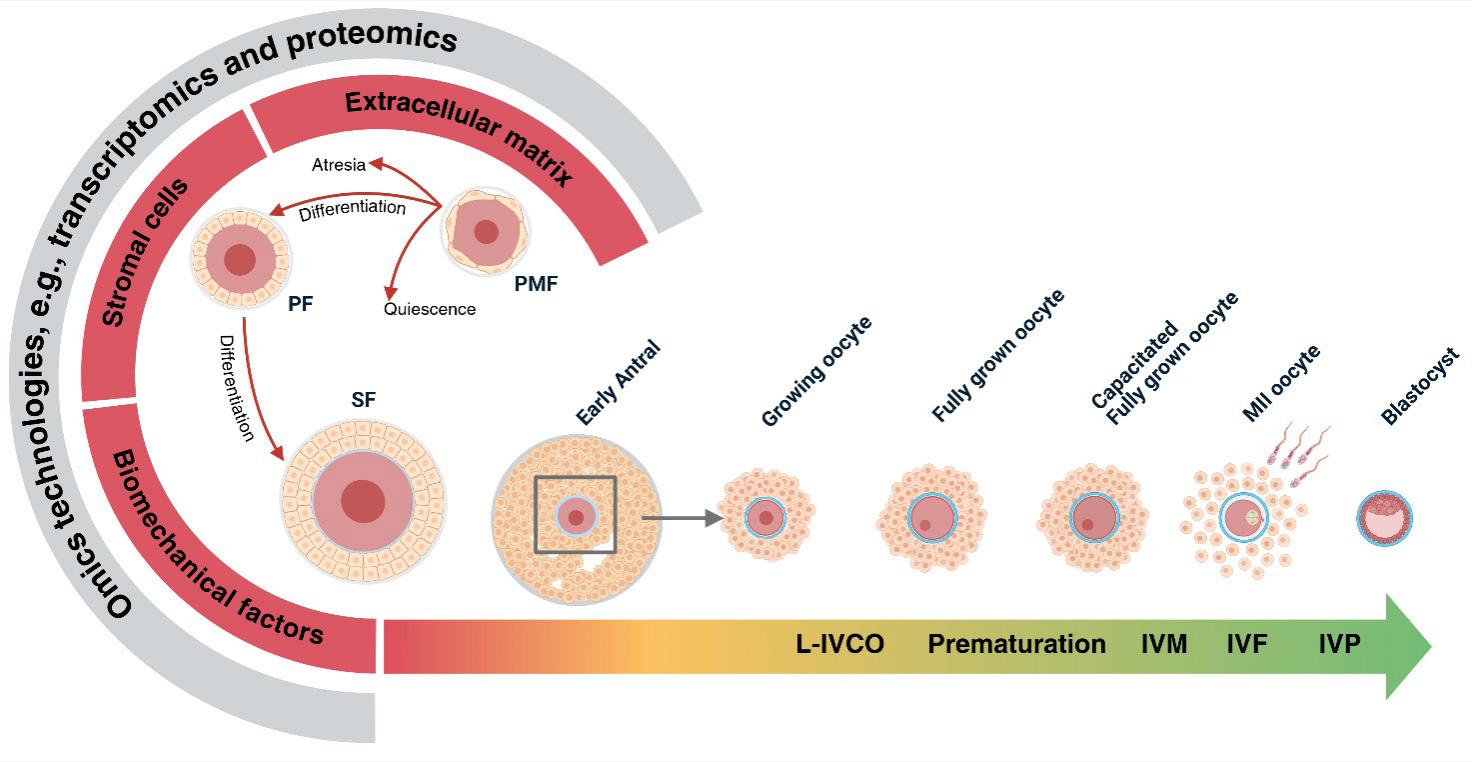
Summarizing the state of ARTs in terms of the feasibility of culture systems throughout folliculogenesis, from PMFs to mature oocytes capable of being fertilized and developing into a blastocyst. The arrow represents the developmental trajectory, with its color indicating the current applicability of in vitro culture at each follicular stage. Red indicates the preantral stage, where no existing culture system can yet fully support follicle development due to the complexity of involved factors. Omics technologies are proposed as a potential strategy to address this limitation. After antrum formation, early antral follicles (yellow) show increasing in vitro culture feasibility, especially bovine and ovine, using long in vitro oocyte culture (L-IVCO) systems, currently limited to a few species but expected applicability to others (Barros et al., 2020; Ebrahimi et al., 2024, 2025). Finally, in green, oocytes undergo a prematuration phase followed by in vitro maturation (IVM), a stage where widely used culture systems are available, enabling the acquisition of full developmental competence for fertilization (IVF) and subsequent in vitro embryo production (IVP) (Fair and Lonergan, 2023; Frost and Gilchrist, 2024; Luciano et al., 2018, 2021; Luciano and Sirard, 2018). Created in BioRender by Monferini (2025).

In our view, advancing in vitro folliculogenesis requires a multidisciplinary approach (Figure 4). By characterizing each stage of the preantral phase as a distinct developmental status with unique requirements, we can gain a deeper understanding of folliculogenesis and optimize the culture system. Additionally, the milieu must consider the mechanisms, including cell-to-cell communication, that guide folliculogenesis, as well as the effect that the stroma may have on follicle differentiation. The dynamic interaction between follicles and stroma, along with the growing body of evidence on mechanotransduction, i.e., the conversion of mechanical cues into molecular signaling, holds promise for enhancing 3D culture systems (Biswas et al., 2022; Matsuzaki, 2021). Together, these remain some of the critical gaps in current in vitro models. To address these issues, future studies should adopt a more integrative perspective, leveraging emerging omics technologies, such as transcriptomics and proteomics, to dissect the complex regulatory networks that guide folliculogenesis.

In conclusion, this review highlights the pivotal role of the ovarian environment in orchestrating folliculogenesis across the reproductive lifespan. The mechanical properties of the extracellular matrix are dynamic, shaped by follicular development, aging, and pathological conditions, and exert a significant influence through mechanotransduction, whereby physical stimuli are translated into biochemical signals that can either promote or inhibit follicular growth. These mechanical changes not only govern the initial activation of follicles but also modulate their differentiation, ovulation, and ultimately, oocyte competence. Crucially, understanding the intricate cross-talk among the cellular and extracellular components of the ovary is essential for guiding the development of in vitro folliculogenesis models. Future studies exploring the 3D architecture and biomechanical landscape of the bovine ovary hold great promise. Such insights will inform the design of biomimetic 3D culture systems capable of recapitulating native tissue dynamics, thereby enhancing our ability to evaluate culture outcomes, engineer functional ovarian constructs, and refine fertility preservation strategies through targeted retrieval of viable gametes.

## Data Availability

Research data is available in the body of the article.
